# Mania after long‐term treatment with daily 10 mg prednisolone

**DOI:** 10.1002/pcn5.66

**Published:** 2022-12-20

**Authors:** Rintaro Fujii, Yuki Konishi, Ryutaro Furusawa, Naomichi Okamoto, Reiji Yoshimura

**Affiliations:** ^1^ Department of Psychiatry University of Occupational and Environmental Health Yahatanishiku Kitakyushu Japan

**Keywords:** corticosteroid, long term, low dose, mania, prednisolone

## Abstract

**Background:**

High‐dose corticosteroids may be accompanied by central nervous system side‐effects, including psychiatric disorders. These psychiatric disorders tend to appear relatively early in treatment. We report an unusual case of mania after long‐term administration of a small dose of prednisolone.

**Case Presentation:**

A patient was treated for relapsed Crohn's disease with a small dose of prednisolone (10 mg/day). After 6 months, she became severely manic. There was no family history of psychiatric disorders. The mania was resistant to olanzapine and sodium valproate, but improved with the reduction of the prednisolone dose. Prednisolone was tapered off while confirming with the gastroenterologist that there was no flare‐up of Crohn's disease. She is now off prednisolone and is doing well, with no outbreaks of Crohn's disease or manic episodes.

**Conclusion:**

This case of severe mania after 6 months of low‐dose prednisolone is unusual. Physicians should be aware that even small doses of long‐term prednisolone may cause the emergence of severe mania.

## Graphical abstract

We present a case of mania after long‐term administration of 10 mg prednisolone q.d. to a patient with a Crohn's disease flare‐up. The mania did not resolve despite treatment with olanzapine and sodium valproate until the prednisolone dose was tapered and discontinued.

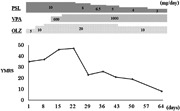

## BACKGROUND

Corticosteroids are frequently used for their anti‐inflammatory and immunosuppressive effects. However, they can be associated with a variety of side‐effects, including psychiatric disorders, because they affect the secretion of neurotransmitters, such as serotonin and dopamine.^1^, ^2^, ^3^ In general, ≥40 mg of prednisolone per day is associated with the appearance of psychiatric disorders.^4^ Previous studies have shown that corticosteroid‑induced psychiatric disorders appear relatively early in treatment, often within a few weeks.^5^, ^6^ We report a case of mania after long‐term administration of a small dose of prednisolone.

## CASE PRESENTATION

A 66‐year‐old woman was admitted to the psychiatry department of our university hospital with a diagnosis of mania. There was no family history of psychiatric disorders. She had had a history of Crohn's disease for 34 years and unmedicated chronic kidney disease (serum creatinine levels, 1.96 mg/dL). Once in the past she had experienced mood elevation during corticosteroid treatment for Crohn's disease. However, its dose and durations were unknown. After a long period of remission, the Crohn's disease had flared up approximately 6 months prior to admission. Prednisolone 10 mg/day was initiated, with resulting remission of the Crohn's disease. After 6 months on this dose of prednisolone, she experienced an abnormally elevated mood, flight of ideas, and decreased need for sleep. There was no personal or family history of substance abuse, diabetes, or thyroid dysfunction.

**Figure 1:**
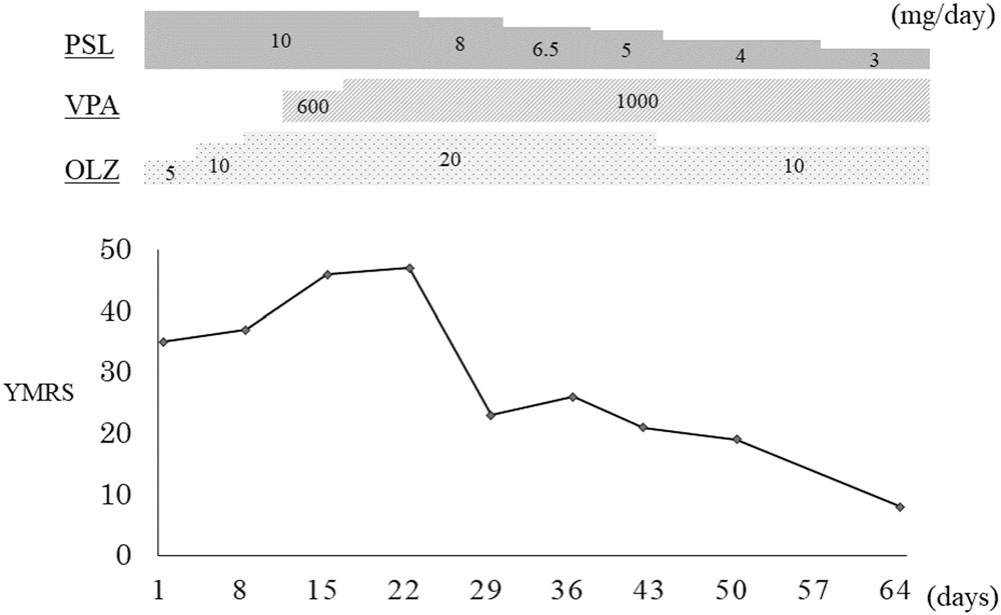
The course of treatment from hospitalization to discharge. OLZ, olanzapine; PSL, prednisolone; VPA, sodium valproate; YMRS, Young Mania Rating Scale.

In the hospital, she showed increased mental activity and agitation. Her memory and orientation were intact, but she exhibited distractibility. She scored 35 on the Young Mania Rating Scale. Head MRI and blood tests showed no abnormalities. The diagnosis of a manic episode was made. Olanzapine was started at 5 mg/day and increased to 20 mg/day over 10 days. Lithium was avoided because of her chronic kidney disease. However, the patient's manic symptoms did not improve after 3 weeks. Therefore, 600 mg of sodium valproate was initiated, and the dose was increased to 1000 mg because the blood concentration of sodium valproate was 41.1 μg/mL (therapeutic range, 50.0–100.0 μg/mL). The manic symptoms did not improve after the dosage increase. Acting on the hypothesis that the low‐dose prednisolone appeared to be a factor in the manic episodes, a diagnosis of manic disorder secondary to corticosteroids was made. The prednisolone was reduced from 10 mg/day to 8 mg/day after gastroenterology confirmed that the Crohn's disease was in remission. The dose was then further tapered down to 6.5, 5, and 4 mg approximately weekly, with caution to avoid flare‐ups of Crohn's disease and the appearance of acute adrenal insufficiency. With tapering, mania improved, and the Young Mania Rating Scale dropped to 8 points (Figure 1). With improvement, olanzapine was tapered, and prednisolone was reduced to 3 mg/day without relapse of mania. The patient was discharged from the psychiatry department. Since then, she has been off prednisolone, sodium valproate, and olanzapine for more than 4 years and is doing well with no flare‐ups of Crohn's disease or manic episodes.

## DISCUSSION AND CONCLUSIONS

This case is noteworthy because the patient presented with a psychiatric disorder after a low dose of prednisolone for 6 months, which differs from the general characteristics of corticosteroid‐induced psychiatric disorders. Olanzapine and sodium valproate were continued during prednisolone tapering off; thus, these effects on treatment for mania were not wholly discounted. However, the mania did not resolve until the prednisolone dose was tapered. Therefore, we suggest the possible causality between small doses and long‐term use of prednisolone and mania in this case.

There are few reported cases of low‐dose corticosteroid‐induced mania. Although the onset of psychiatric symptoms has been suggested to correlate with the dose of prednisolone, psychiatric symptoms can occur even at low doses.^7^ In a previous report by Hong et al., a small dose of prednisolone caused agitation in a patient on the first day of administration.^8^ Unlike that case, our present patient developed mania after long‐term administration. With long‐term administration, 55% of patients have reported mood disturbances after 6 months of low‐dose prednisolone.^9^ Except for one case of mixed features, these mood disorders were all depressive episodes, and there were no pure manic episodes. Therefore, to our knowledge, severe manic episodes after long‐term administration of low‐dose prednisolone are infrequent.

It has been reported that corticosteroids increase the risk of recurrence of pre‐existing mood disorders.^10^ This patient's past presentation with elevated mood may have been a risk factor for her mania. Another possibility was that her Crohn's disease activity change may have influenced the manic symptoms because of the elevated prevalence of psychiatric disorders seen in inflammatory bowel disease.^11^ The use of corticosteroid treatment has been reported to be a significant risk factor for mood disorders in inflammatory bowel disease patients.^12^ Since there was no correlation between inflammatory bowel disease activity change and mood change in this patient, we suggest that corticosteroids, rather than the relapse of Crohn's disease, led to the mania in this case. In contrast, it is difficult to conclude that chronic kidney disease is a risk factor for corticosteroid‑induced psychiatric disorders. The pharmacokinetics and pharmacodynamics of prednisolone in renal impairment are unclear.^13^, ^14^, ^15^ There are also reports of decreased excretion rates of prednisolone in uremic patients,^16^ but in the present case, uremia was not present. A causal relationship between the risk of corticosteroids‐induced psychiatric disorders and renal dysfunction is not known. Additionally, we did not measure plasma levels of adrenocorticotropic hormone (ACTH) during daily 10 mg of prednisolone. Had plasma ACTH levels been suppressed, 10 mg of prednisolone might have been considered high enough for this patient.

In this case, the patient's Crohn's disease activity was stabilized, which made it possible to proceed with the downward titration and eventual discontinuation of prednisolone. We believe it is essential for psychiatrists to discuss the treatment plan for possible corticosteroid‑induced psychiatric disorders with internists and to develop a treatment plan for both the underlying inflammatory disease and the psychiatric disorders.

In conclusion, even small doses and long‐term use of prednisolone may be a possible association with the emergence of severe mania.

## AUTHOR CONTRIBUTIONS

Rintaro Fujii, Ryutaro Furusawa, and Naomichi Okamoto treated the patient. Rintaro Fujii, Yuki Konishi, Ryutaro Furusawa, and Reiji Yoshimura wrote the draft. All authors reviewed the draft and revised the manuscript.

## DISCLOSURE STATEMENT

The authors declare no conflict of interest.

## ETHICS APPROVAL STATEMENT

A signed release and verbal informed consent were obtained from the patient before reporting this case.

## PATIENT CONSENT STATEMENT

A signed release and verbal informed consent were obtained from the patient before reporting this case.

## CLINICAL TRIAL REGISTRATION

N/A.

## Data Availability

N/A.

## References

[1] Schäcke H , Döcke WD , Asadullah K . Mechanisms involved in the side effects of glucocorticoids. Pharmacol Ther. 2002;96:23–43. 12441176 10.1016/s0163-7258(02)00297-8

[2] Patten SB , Williams JVA , Love EJ . Self‐reported depressive symptoms following treatment with corticosteroids and sedative‐hypnotics. Int J Psychiatry Med. 1996;26:15–24. 8707452 10.2190/BL97-BWFR-4QR0-CEY7

[3] Dubovsky AN , Arvikar S , Stern TA , Axelrod L . The neuropsychiatric complications of glucocorticoid use: steroid psychosis revisited. Psychosomatics. 2012;53:103–15. 22424158 10.1016/j.psym.2011.12.007

[4] Collaborative Drug Surveillance B . Acute adverse reactions to prednisone in relation to dosage. Clin Pharm Ther. 1972;13:694–8. 10.1002/cpt1972135part1694 5053810

[5] Lewis DA , Smith RE . Steroid‐induced psychiatric syndromes. J Affect Disord. 1983;5:319–32. 6319464 10.1016/0165-0327(83)90022-8

[6] Hall TC , Choi OS , Abadi A , Krant MJ . High‐dose corticoid therapy in Hodgkin's disease and other lymphomas. Ann Intern Med. 1967;66:1144–53. 5338940 10.7326/0003-4819-66-6-1144

[7] Kenna HA , Poon AW , de los Angeles CP , Koran LM . Psychiatric complications of treatment with corticosteroids: review with case report. Psychiatry Clin Neurosci. 2011;65:549–60. 22003987 10.1111/j.1440-1819.2011.02260.x

[8] Hong SI , Cho DH , Kang HC , Chung DJ , Chung MY . Acute onset of steroid psychosis with very low dose of prednisolone in Sheehan's syndrome. Endocr J. 2006;53:255–8. 16618985 10.1507/endocrj.53.255

[9] Bolanos SH , Khan DA , Hanczyc M , Bauer MS , Dhanani N , Brown ES . Assessment of mood states in patients receiving long‐term corticosteroid therapy and in controls with patient‐rated and clinician‐rated scales. Ann Allergy Asthma Immunol. 2004;92:500–5. 15191017 10.1016/S1081-1206(10)61756-5

[10] Fardet L , Petersen I , Nazareth I . Suicidal behavior and severe neuropsychiatric disorders following glucocorticoid therapy in primary care. Am J Psychiatry. 2012;169:491–7. 22764363 10.1176/appi.ajp.2011.11071009

[11] Bernstein CN , Hitchon CA , Walld R , Bolton JM , Sareen J , Walker JR , et al. Increased burden of psychiatric disorders in inflammatory bowel disease. Inflamm Bowel Dis. 2019;25:360–8. 29986021 10.1093/ibd/izy235 PMC6391845

[12] Ou G , Bressler B , Galorport C , Lam E , Ko HH , Enns R , et al. Rate of corticosteroid‐induced mood changes in patients with inflammatory bowel disease: a prospective study. J Can Assoc Gastroenterol. 2018;1:99–106. 31294728 10.1093/jcag/gwy023 PMC6507281

[13] Bergmann TK , Barraclough KA , Lee KJ , Staatz CE . Clinical pharmacokinetics and pharmacodynamics of prednisolone and prednisone in solid organ transplantation. Clin Pharmacokinet. 2012;51:711–41. 23018468 10.1007/s40262-012-0007-8

[14] Czock D , Keller F , Rasche FM , H??ussler U . Pharmacokinetics and pharmacodynamics of systemically administered glucocorticoids. Clin Pharmacokinet. 2005;44:61–98. 15634032 10.2165/00003088-200544010-00003

[15] Honoré PM , Jacobs R , De Waele E , De Regt J , Rose T , Van Gorp V , et al. What do we know about steroids metabolism and “PK/PD approach” in AKI and CKD especially while on RRT ‐ current status in 2014. Blood Purif. 2014;38:154–7. 25471548 10.1159/000368390

[16] Bergrem H . The influence of uremia on pharmacokinetics and protein binding of prednisolone. Acta Med Scand. 1983;213:333–7. 6880855 10.1111/j.0954-6820.1983.tb03747.x

